# Temporal patterns of genetic variation in a salmon population undergoing rapid change in migration timing

**DOI:** 10.1111/eva.12066

**Published:** 2013-04-18

**Authors:** Ryan P. Kovach, Anthony J. Gharrett, David A. Tallmon

**Affiliations:** ^1^ Biology and Wildlife Department Institute of Arctic Biology University of Alaska Fairbanks Fairbanks AK USA; ^2^ School of Fisheries and Oceanic Sciences University of Alaska Fairbanks Juneau AK USA; ^3^ Biology and Marine Biology Program University of Alaska Southeast Juneau AK USA

**Keywords:** climate change, genetic change, genetic divergence, genetic diversity, genetic effective population size, phenology, salmon

## Abstract

Though genetic diversity is necessary for population persistence in rapidly changing environments, little is known about how climate‐warming influences patterns of intra‐population genetic variation. For a pink salmon population experiencing increasing temperatures, we used temporal genetic data (microsatellite = 1993, 2001, 2009; allozyme = 1979, 1981, 1983) to quantify the genetic effective population size (*N*_*e*_) and genetic divergence due to differences in migration timing and to estimate whether these quantities have changed over time. We predicted that temporal trends toward earlier migration timing and a corresponding loss of phenotypic variation would decrease genetic divergence based on migration timing and *N*_*e*_. We observed significant genetic divergence based on migration timing and genetic heterogeneity between early‐ and late‐migrating fish. There was also some evidence for divergent selection between early‐ and late‐migrating fish at circadian rhythm genes, but results varied over time. Estimates of *N*_*e*_ from multiple methods were large (>1200) and *N*_*e*_/*N*_*c*_ generally exceeded 0.2. Despite shifts in migration timing and loss of phenotypic variation, there was no evidence for changes in within‐population genetic divergence or *N*_*e*_ over the course of this study. These results suggest that in instances of population stability, genetic diversity may be resistant to climate‐induced changes in migration timing.

## Introduction

Describing and understanding the distribution of genetic variation within populations is fundamental to the management of species, particularly in a rapidly changing world (Allendorf and Luikart 2007). Climate‐induced changes in the spatial distribution and phenology of populations can influence numerous aspects of demography including dispersal, survival, reproductive success, and overall abundance, all of which have consequences for the distribution of genetic variation within and among populations (Frankham 1996; Parmesan 2006; Pauls et al. 2012). For example, reductions in habitat and increasing fragmentation as a result of distributional shifts toward higher elevation can reduce genetic diversity within and increase genetic divergence among populations of alpine mammals (Rubidge et al. 2012). Similarly, phenological changes – changes in the seasonal timing of life history events such as migration – could alter patterns of genetic diversity for populations that exhibit intra‐population genetic divergence based on differences in phenology (Hendry and Day 2005; Heard et al. 2012), Changes in phenology may also influence variability in reproductive success for those populations where phenology directly influences individual fitness. Despite substantial evidence for climate‐induced changes in phenology (Parmesan 2006), there is little information documenting how these changes influence microevolution within populations (Franks and Weiss 2009; Heard et al. 2012).

**Figure 1:**
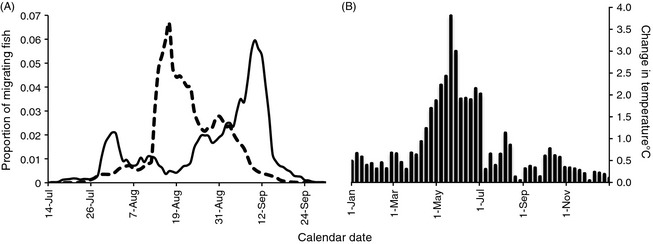
The intra‐annual distribution of migration timing (reproductive timing) and stream temperature in Auke Creek Alaska. The lines in panel (A) are the 5‐day running averages of the proportion of odd‐year pink salmon migrating into Auke Creek averaged from 1971 to 1979 (solid line) and 2003–2011 (dashed line). Panel (B) depicts the difference in °C between the average weekly stream temperatures from 2001 to 2010 and 1971 to 1980 (i.e. mean weekly stream temperature (2001–2010) – mean weekly stream temperature (1971–1980)).

To determine how phenological changes can influence intra‐population genetic diversity, we focused on a pink salmon (*Oncorhynchus gorbuscha*) population in rapidly warming (Fig. 1A) Auke Creek, Alaska. This population now migrates into freshwater to reproduce approximately 2 weeks earlier than in 1971 and has lost nearly 30% of its phenotypic variation in migration timing (Fig. 1B, Taylor 2008; Kovach et al. 2012a, 2013). Auke Creek pink salmon reproduce soon after entering freshwater; consequently, migration timing marks the beginning of reproduction for each individual fish. Within this population, adult migration timing is heritable, there is a genetic component to developmental rates, and there is evidence for local adaptation based on migration timing for a suite of life‐history traits (Hebert et al. 1998; Smoker et al. 1998). Changes in migration timing for this population appear to be due, at least in part, to microevolutionary responses to natural selection against late‐migrating fish (Kovach et al. 2012a). Although the exact causal mechanisms of the phenotypic and evolutionary changes are unknown, several lines of evidence suggest that these shifts are due to climate warming. There is strong evidence of a genetic change toward an increasing prevalence of the early‐migrating phenotype after an exceptionally warm year (Kovach et al. 2012a), and there have been widespread shifts toward earlier migration timing in several salmonid populations (including the even‐year pink salmon population) at this location and elsewhere (Kovach et al. 2013). The shift toward earlier migration timing for this population might actually be due to selection for earlier migration timing in juvenile pink salmon (Kovach 2012b), a trait that is directly influenced by adult migration timing (Smoker et al. 1998; Taylor 2008). Importantly, the phenotypic changes in this population do not appear to be due harvest or hatchery influences (Kovach et al. 2013). Thus, this population is ideal for exploring how climate‐induced changes in reproductive timing can influence genetic diversity.

Ultimately, the ability to adapt to novel environmental conditions is limited by the amount of genetic diversity within a population (Frankham 1995a; Allendorf and Luikart 2007). Loss of genetic diversity can increase probability of extinction because genetic variability gives rise to alternative phenotypes (e.g. morphologies or behaviors) that can respond to environmental change (Lacy 1997; Frankham 2005). At a larger scale, genetic diversity can influence ecological interactions within and between species, and thereby impact overall ecosystem dynamics (Hughes et al. 2008; Palkovacs et al. 2011), making it a critical component of biodiversity which merits further attention in conservation and natural resource management (Laikre 2010).

One way to measure a population's evolutionary potential and genetic diversity is the genetic effective size of a population (*N*_*e*_). The *N*_*e*_ of a population is one of the most important parameters in evolutionary and conservation biology (Waples 1989; Frankham 1995b) because it describes the rate at which genetic variation is lost, the influence of inbreeding, and the relative strengths of selection and migration in determining allele frequencies (Allendorf and Luikart 2007). In so doing, *N*_*e*_ provides important information about population viability (Frankham 1995b). Many factors can cause a population's *N*_*e*_ to be less than the census population size (*N*_*c*_) including natural selection, uneven sex ratios, temporal variation in population size, that exceed Poisson variance in reproductive success, and population age structuring (Frankham 1995b). As such, *N*_*e*_ is a particularly useful parameter because it captures information about genetic and demographic processes.

Little is known about *N*_*e*_ and the *N*_*e*_ to *N*_*c*_ ratio for pink salmon. Pink salmon have approximately equal sex ratios and non‐overlapping generations; therefore, variance in reproductive success (Geiger et al. 1997) and inter‐generational fluctuations in population abundance (Kalinowski and Waples 2002) should be the primary factors that reduce *N*_*e*_ relative to *N*_*c*_ for this species. Variance in the reproductive success of pink salmon may exceed Poisson (over‐dispersed) because competition for spawning areas (i.e., density dependence) can lead to redd superimposition (i.e. destruction of spawning redds and reproductive failure of some adults; Groot and Margolis 1991; Fukushima et al. 1998; Quinn 2005). Additionally, pink salmon populations, including those in Auke Creek, can have family‐correlated marine survival (Geiger et al. 1997, 2007), which further inflates variance in reproductive success because a few families have very high survival while many others have low survival (i.e. do not replace themselves). Whether phenological changes in Auke Creek pink salmon have influenced *N*_*e*_ is unknown. Changes in migration timing could influence *N*_*c*_ over time by altering variability in reproductive success as a result of natural selection against late‐migrating fish, and/or by increasing density dependence owing to a compressed distribution of reproductive timing (i.e. more fish are now spawning over a shorter period of time).

Describing genetic population structure within‐ and between‐populations is another way to quantify genetic diversity. Understanding within‐ and between‐population genetic structure is critical to understanding the evolutionary and demographic forces influencing a population and for making informed management decisions (Waples 1998; Waples and Gaggiotti 2006). Whereas genetic structure between populations is a well‐described phenomenon, much less attention has been given to within‐population genetic structure resulting from phenotypic differences among individuals (Hendry and Day 2005). As a result of high heritability in migration and reproductive timing (median *h* ^2^ = 0.51, Carlson and Seamons 2008), salmonid populations often exhibit significant intra‐annual genetic divergence based on reproductive timing (McGregor et al. 1998; Fillatre et al. 2003; Hendry and Day 2005). The Auke Creek pink salmon population historically exhibited temporal population structuring, including genetic divergence between early‐ and late‐migrating fish, at selectively neutral loci (McGregor et al. 1998) and an experimental genetic marker (Lane et al. 1990; Gharrett et al. 2001; Kovach et al. 2012a). For salmonid fishes, researchers have identified several circadian rhythm genes that are related to migration timing itself, or traits that influence migration timing (e.g. development rate; O'Malley et al. 2010b; O'Malley and Banks 2008; O'Malley et al. 2010a,b). This offers an opportunity to compare patterns of intra‐population genetic diversity at circadian rhythm genes with patterns at selectively neutral genes. If the circadian rhythm genes are partially responsible for the heritability (i.e. additive genetic variance) in migration timing for pink salmon in this population (Smoker et al. 1998), genetic divergence at the circadian rhythm genes should exceed differentiation at neutral genes (Nosil et al. 2009).

We hypothesized that changes in migration timing in the Auke Creek pink salmon population could influence intra‐population genetic diversity through several mechanisms. First, loss of divergence between early‐ and late‐migrating fish could arise via genetic admixture as a result of a compressed spawning distribution, and/or as a result of decreased genetic variation due to a strong reduction in the late‐migrating phenotype (i.e. truncation of the migration timing distribution). Alternatively, increases in divergence are possible if genetic drift was increased among late‐migrating fish because of a decrease in the abundance of this phenotype_._ Finally, changes in migration timing could increase variance in reproductive success as a result of natural selection against late‐migrating fish and by increasing density dependence, both of which may act to decrease *N*_*e*_ over time.

Specifically, we addressed four objectives related to the genetic diversity of Aukc Creek pink salmon: (i) describe patterns of genetic divergence and temporal autocorrelation in allele frequencies due to variation in migration timing, (ii) determine if circadian rhythm genes appear to be related to migratory timing, (iii) estimate *N*_*e*_ and the *N*_*e*_ to *N*_*c*_ ratio, and (iv); test if *N*_*e*_ and genetic divergence based on migration timing have changed over time. These objectives clarify how genetic diversity is distributed in salmonid populations and directly test our hypothesis that shifts in migration timing can alter patterns of intra‐population genetic diversity.

## Methods

### Study site, population and genetic data

Pink salmon have a strictly semelparous, 2‐year life cycle that produces distinct odd‐ and even‐year populations within a stream (Aspinwall 1974), all individuals migrate to the ocean prior to maturation (i.e. no fish mature in freshwater). This study focuses on the odd‐year pink salmon population, which has been censused at a permanent weir structure during its spawning migration into Auke Creek, Alaska, since 1971. The permanent weir structure is located directly above the high tide mark and therefore this study focuses on individuals that migrate into freshwater. Importantly, a small segment of the population does spawn in the intertidal area below the weir, but gene flow between these life histories/populations appears to be restricted (Gharrett et al. 2001; Gilk et al. 2004). Pink salmon migrate into Auke Creek from early August until the end of September and their median date of migration timing (at present) tends to occur from August 20–25. Early‐ and late‐migrating fish generally use the same habitats for spawning and as a result there is evidence for competition between fish for spawning locations (Fukushima et al. 1998; Smoker et al. 1998). From 1971 to 2011 the abundance of pink salmon varied widely in Auke Creek, from *N*_*c*_ *=* 1548 (1995) to *N*_*c*_ = 28 127 (1999), but the population is stable and population growth rate is at the replacement level (λ ≈ 1.0, Kovach et al. 2013). Tissue samples that had been archived were analyzed for this study, in conjunction with genetic data from another study of this population that took place from 1979 to 1983 (McGregor 1983; McGregor et al. 1998).

Fish were sampled as they migrated through the Auke Creek weir (2001 and 2009) or from recent (<24 h) carcasses (1993). Genetic samples were collected from 10 fish every other day so that 170–192 fish were genotyped in each year (See Table 1 for sample sizes used for each analysis). Each fish was genotyped at 23 microsatellite loci, three of which (*OtsClock1b*,* Cry2b, Cry3*) are located within the *Clock* and *Cryptochrome* circadian rhythm genes that that are correlated with migration timing and development rate in several salmonid species and populations (O'Malley and Banks 2008; O'Malley et al. 2010a,b). Additionally, there is a marginally significant geographical cline in *OtsClock1b* frequencies in pink salmon, indicating that this locus may be related to migration timing in this species (O'Malley et al. 2010a). Complete descriptions of tissue sampling and microsatellite genotyping were presented in Kovach et al. (2012a). We checked for deviations from Hardy–Weinberg predictions by using a pseudo‐exact test and tested for significant pair‐wise linkage disequilibrium between loci in GENEPOP (Raymond and Rousset 1995).

**Table 1:** Sample sizes (number of individuals or genotypes across loci) used for each of the genetic analyses in each year. Timing refers to the period that genetic samples were collected from the intra‐annual migration timing distribution. For example, ‘early’ refers to the number of samples collected from the earliest migrating fish, while ‘combined’ refers to the total number of samples across the entire migration timing distribution. Italicized values are the harmonic mean number of genotypes at each locus (as opposed to number of individuals sampled)

Analysis/method	Year	Timing			
*G”*_*ST*_ and Lositan (outlier test)		Early	Late		
	1979	*80.66*	*97.03*		
	1981	*100.63*	*101.45*		
	1983	*90.04*	*125.07*		
	1993	*48.96*	*62.89*		
	2001	*61.95*	*49.43*		
	2009	*44.94*	*46.80*		
G‐tests (homogeneity tests)		1st quartile	2nd quartile	3rd quartile	4th quartile
	1993	*48.81*	*57.63*	*36.46*	*41.30*
	2001	*95.58*	*9.25*	*29.78*	*89.04*
	2009	*44.86*	*18.64*	*46.54*	*95.65*
STRUCTURE and genetic autocorrelation		Combined			
	1993	170			
	2001	189			
	2009	192			
*N*_*e*_ estimates		Combined			
	1993	148			
	2001	182			
	2009	178			

### Data analysis

#### Genetic structure based on migration timing

We calculated *G”*_*ST*_ (Meirmans and Hedrick 2011) between the earliest and latest migrating fish in 1979, 1981, 1983, 1993, 2001, and 2009. Estimates of *G”*_*ST*_ from 1979 to 1983 were based on allele frequencies from 10 to 11 allozyme loci (McGregor 1983; McGregor et al. 1998). Sample sizes varied between loci, run components (early or late), and year, but averaged approximately 100 for both early and late migrating fish from 1979 to 1983, and approximately 50 for both early‐ and late‐ migrating fish from 1993 to 2009. We used *G”*_*ST*_ as our measurement of effect size because it is relatively insensitive to the substantial differences between allozyme and microsatellite loci in mutation rates and the numbers of alleles (Hedrick 2005; Meirmans and Hedrick 2011). For the microsatellite data, we used GenoDive (Meirmans and Van Tienderen 2004) to calculate *G”*_*ST*_ and associated 95% confidence intervals by bootstrapping over loci. Because we did not have genotypic data (only allele frequencies and sample sizes) for the allozymes, we calculated *G”*_*ST*_ manually and obtained 95% confidence intervals by bootstrapping over loci in the ‘boot’ package in Program R (R Development Core Team [Link]). To test the hypothesis that genetic divergence based on migration timing has declined as a result of changes toward earlier migration timing and decreasing phenotypic variation, we compared 95% confidence intervals for *G”*_*ST*_ between years. This method is more conservative than directly testing for a significant difference between two estimates; but with large numbers of molecular markers, this is a powerful method to detect genetic change in a population (Schwartz et al. 2007).

We used multiple methods to describe within‐population genetic structure for genotypes collected from 1993, 2001, and 2009. Temporal genetic autocorrelation based on migration timing was estimated using GENALEX V. 6.3 (Smouse and Peakall 1999; Peakall and Smouse 2006). If temporal population structure exists within a population, the genetic correlation between individuals decreases as the time period between dates of migration timing increases. Specifically, this method condenses the genetic data from the microsatellite loci into a matrix of pair‐wise individual‐by‐individual squared genetic distances (Smouse and Peakall 1999) in order to compute correlation coefficients between groups of individuals. We used 4‐day periods as our distance class (grouped individuals that migrated within 4 days of each other), and tested for autocorrelation as a function of the number of days between samples (Peakall et al. 2003). We also investigated the influence of grouping individuals for other distance classes (1 and 2 days) but it had little quantitative or qualitative effect. For each year, we used 9999 permutations and 999 bootstrap replicates to estimate variance and assess significance. We compared across years the 95% confidence intervals for the correlation coefficients estimated in 1993, 2001, and 2009 to test for inter‐annual changes in genetic population structure based on migration timing.

Population genetic structure of pink salmon within Auke Creek may exist along a gradient of time (isolation by time) or bimodally/multimodally (i.e. an early and late migrating population). To test for distinct population groupings we used the program STRUCTURE (Pritchard and Rosenberg 1999) to estimate the number of sub‐populations (*K*) within the overall migration timing distribution. For each year we used the admixture model, no prior information about population of origin, 100 000 iterations of burn‐in, and 500 000 samples from the posterior distribution to estimate the likelihood of *K* given the data. We considered *K *= 1–6 and averaged the log‐likelihood based on four iterations of the MCMC chain.

We used *G*‐tests for genic divergence in GENEPOP (Raymond and Rousset 1995) to test directly for genetic homogeneity between non‐consecutive quartiles of the migration timing distribution. Quartiles of the migration timing distribution were determined from the census of migrating pink salmon collected at Auke Creek. Samples collected on the day that a particular quartile was reached (e.g. 25 percentile) were allocated to both the first and second quartile. Consequently, we did not test for divergence between adjacent quartiles.

To determine if the circadian rhythm loci *OtsClock1b, Cry2*, and *Cry3* are related to migration timing in this population, we used two approaches. Because allele length at *Clock* genes has been shown to be related to phenological traits in several bird populations (Liedvogel et al. 2009; Caprioli et al. 2012), we regressed the total allele lengths (length of allele 1 + length of allele 2) of each individual versus date of migration timing for each locus in each year (Liedvogel et al. 2009). We also used an *F*_*ST*_ outlier approach (Beaumont and Nichols 1996) to test if there has been selection at the three circadian rhythm loci or any of the putatively neutral loci. Larger than expected values of genetic differentiation provide evidence that a locus is under selection and therefore contributes to, or is linked to genes influencing, the phenotype (Nosil et al. 2009). Data from the first and last 10 days of sampling were used to represent the ‘early’ and ‘late’ migrating phenotypes, respectively. For each year, we used LOSITAN (Antao et al. 2008) to test if divergence (*F*_*ST*_) between early‐ and late‐migrating fish at any particular locus differed from a null distribution of *F*_*ST*_ generated from the empirical data assuming an island model of gene flow between early‐ and late‐migrating fish.

#### *N*_*e*_ and the *N*_*e*_/*N*_*c*_ ratio

*N*_*e*_ was estimated from the temporal variance in allele frequencies across samples (*F*_*TEMP*_ and MLNe), linkage disequilibrium within a sample (LDNe), and approximate Bayesian computation based on summary statistics estimated from single samples (ONeSAMP). We used multiple approaches because each method makes different use of the data and in some cases estimates conceptually different values (inbreeding versus variance effective population sizes), thereby providing a more robust understanding of *N*_*e*_ and the *N*_*e*_/*N*_*c*_ ratio (Luikart et al. 2010; Waples and Do 2010). This let us better evaluate if *N*_*e*_ and *N*_*e*_/*N*_*c*_ have changed from 1993 to 2009.

The *F*_*TEMP*_ approach requires genetic samples from at least two time periods and estimates *N*_*e*_ based on the value of *N*_*e*_ that would generate the observed genetic differences between samples (Waples 1989). Samples were available from three time periods, which made it possible to make three *N*_*e*_ estimates (1993–2001, 2001–2009, 1993–2009). NEESTIMATOR 1.3 was used to estimate *N*_*e*_ with the *F*_*TEMP*_ approach (Peel et al. 2004). Similarly, MLNe requires genetic samples from multiple points in time, but uses a maximum likelihood approach to estimate *N*_*e*_ (as opposed to the moments‐based *F*_*TEMP*_; Wang 2001; Wang and Whitlock 2003). We used the same‐paired samples to estimate *N*_*e*_ with MLNe (e.g. 1993–2001), except MLNe also makes use of the 2001 sample for the 1993–2009 estimate of *N*_*e*_. The upper bound for *N*_*e*_ was 10 000.

Two single sample approaches were used to estimate *N*_*e*_. We used the program LDNe (Waples and Do 2008) to estimate *N*_*e*_ using the linkage disequilibrium approach (Waples 2006) and report estimates based on excluding alleles with frequency <0.02 (Waples and Do 2010). We also estimated *N*_*e*_ with ONeSAMP, which estimates *N*_*e*_ by making use of eight population genetics summary statistics and compares the observed estimates of the summary statistics to values obtained from simulated Wright‐Fisher populations of known *N*_*e*_ (Tallmon et al. 2004; ; Tallmon et al. 2008). For the prior distribution on *N*_*e*_ we used 100–3000. Data at circadian rhythm genes were not used to estimate *N*_*e*_, so a total of 20 loci were used. ONeSAMP requires that individuals have data at more than 75% of loci. After removing individuals that did not have genotypic data at >75% of loci, we retained 148 (1993), 182 (2001), and 178 (2009). This filtered data set was used to estimate *N*_*e*_ for each method (*F*_*TEMP*_, MLNe, LDNe, ONeSAMP).

To calculate *N*_*e*_/*N*_*c*_ based on results from ONeSAMP and LDNe, we used the *N*_*c*_ value from the generation prior to the *N*_*e*_ estimate (Waples 2005; Palstra and Fraser 2012). To calculate *N*_*e*_/*N*_*c*_ based on *N*_*e*_ values from the *F*_*TEMP*_ and MLNe method, we used the harmonic mean of *N*_*c*_ for the time period spanning from the first sample collection to the generation prior to the second sample collection (Waples 2005). Non‐overlapping confidence/credible intervals of *N*_*e*_ provide evidence that these values have changed during the time period of this study (Schwartz et al. 2007).

## Results

### Genetic data

We genotyped Auke Creek odd‐year pink salmon at 23 microsatellite loci in 1993, 2001, and 2009. All of the loci conformed to Hardy–Weinberg expectations and/or had *F*_*IS*_ values near zero for at least two of the years (i.e. no evidence of null alleles), except for two circadian rhythm genes (*OtsClock1b*,* Cry3*) that had elevated *F*_*IS*_ values in one or more years. Nonetheless, we retained these loci in analyses because we *a priori* predicted there might be some divergent selection at these genes. Given 23 loci, there were 253 pair‐wise tests for linkage disequilibrium in each year, and by chance we expected to observe 13 significant values (at α < 0.05). In each year, the number of significant estimates was ≤13 (1993 = 12, 2001 = 9, 2009 = 13). No pairs of loci exhibited significant linkage in all 3 years.

### Genetic structure by migration timing

**Figure 2:**
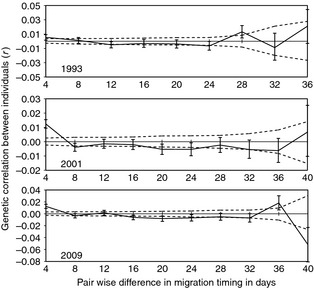
Genetic autocorrelation (*r*) as a function of the number of days between samples from the migration timing distribution. The solid black line is the point estimate for *r* relative to the number of days between migration dates. Dashed lines denote the permutation based 95% confidence areas, and error bars for the point estimates are 95% bootstrap confidence intervals.

Intra‐generational estimates of *G”*_*ST*_ between the earliest and latest migrating fish ranged from −0.002 to 0.011 for data from 1979 to 2009, but bootstrap 95% confidence intervals included 0 in each year for which we had data (Table 2). In 1993, 2001, and 2009 there was evidence of significant (*P* < 0.05) positive autocorrelation (*r *= 0.005 in 1993; *r = *0.012 in 2001; *r *= 0.013 in 2009) between individuals migrating within 4 days of one another (Fig. 2). The majority (20 of 26) of estimates for *r* were negative for fish that migrated more than 4 days apart from one another, meaning that individuals migrating at different times differ genetically more than would be expected by chance. The largest single estimate (*r* = −0.052 CI: −0.0231–−0.0808) was for the maximum distance class (40 day separation) in 2009. Weak negative autocorrelation was significant (*P *< 0.05) in 14 of the 20 estimates before a sequential Bonferroni correction for multiple tests, and significant in 7 of 20 after correction (each data set was corrected independently).

**Table 2:** Estimates of *G”*_*ST*_ between early and late migrating fish from 1979 to 2009. LCI is the lower 95% bootstrap confidence interval, and UCI is the upper 95% bootstrap confidence interval

Year	*G”*_*ST*_	LCI	UCI
1979	−0.001	−0.006	0.002
1981	0.011	−0.003	0.023
1983	0.004	−0.003	0.010
1993	−0.002	−0.007	0.003
2001	0.005	−0.002	0.015
2009	0.002	−0.006	0.012

There was no evidence of population clustering or substructure revealed by STRUCTURE (i.e. *K* = 1). Similarly, *G‐*tests for homogeneity failed to detect genetic divergence (*P > *0.05) between quartiles of the migration timing distribution in 1993 (Table 3). However, *G‐*tests based on data from 2001 showed significant (*P *< 0.05) divergence between fish sampled in the first and third quartile of the migration timing distribution into Auke Creek, and between fish sampled in the first and fourth quartiles. In 2009, there was significant genetic divergence between fish from the first and fourth quartiles of the migration distribution, and between fish from the second and fourth quartiles.

**Table 3:** Results (*P‐*values) for *G*‐tests for genetic divergence between non‐consecutive quartiles of the migration timing distribution. 1, 2, 3, and 4 refer to the first, second, third, and fourth quartiles of the migration timing distribution in each year. Bold values are significant after correction for multiple tests

Quartile	1993		2001		2009	
	1	2	1	2	1	2
3	0.078		**0.002**		0.088	
4	0.782	0.369	**0.003**	0.912	**0.034**	**0.009**

**Figure 3:**
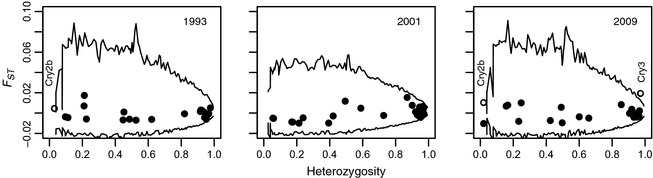
Genetic outlier tests for detecting selection at the circadian rhythm and putatively neutral microsatellite loci. *F*_*ST*_ values are between early‐ and late‐migrating fish. The circles are the point estimates of *F*_*ST*_ for each locus. The black lines denote the neutral 95% confidence intervals for *F*_*ST*_ (i.e. values within the black lines can be explained by genetic drift). Each *F*_*ST*_ outlier is labeled.

Across all years, allelic richness for *OtsClock1b, Cry2b,* and *Cry3* was 5.78, 2.17, and 42.57, and heterozygosity was 0.175, 0.053, and 0.896 respectively. There were no significant relationships between allele lengths and the date of migration for any of the circadian rhythm loci in any year. Three values of *F*_*ST*_ between early‐ and late‐migrating fish exceeded neutral expectation (α = 0.05), and in each instance it was one of the loci associated with circadian rhythm genes. However, the outlier loci apparently under directional selection between early‐ and late‐migrating individuals differed between temporal samples (Fig. 3). Specifically, the loci exhibiting higher *F*_*ST*_ values than neutral expectations were *Cry2b* (*F*_*ST*_ = 0.005) in 1993 and *Cry2b* (*F*_*ST*_ = 0.010) and *Cry3* (*F*_*ST*_ = 0.019) in 2009, while no locus was found in this category in 2001. Interestingly, the largest *F*_*ST*_ value in 1993 was for *OtsClock1b* (*F*_*ST*_ = 0.017), but this value did not exceed neutral expectation. With 69 *F*_*ST*_ estimates (across all years), we would anticipate approximately three false positives (69 × 0.05) at α = 0.05, so these results should be interpreted with caution. Nevertheless, it is notable that the only loci that demonstrated outlier behavior were the circadian rhythm genes that we considered *a priori* to be candidates for natural selection.

Because there were no consistent signals for selection at the circadian rhythm genes, nor evidence that allele lengths were related to migratory timing, estimates of genetic divergence used data at the candidate loci. However, removing the candidate loci resulted in just one fewer significant autocorrelation value (i.e. 13 significant values instead of 14), and the *G*‐test for divergence between the first and fourth quartile in 2009 became non‐significant (*P = *0.19). All other results were qualitatively identical.

### Genetic effective population size and N_e_/N_c_ ratio

Point estimates of *N*_*e*_ based on *F*_*TEMP*_ ranged from 1079 to 3788 depending on the time period of interest, and all lower confidence/credible intervals exceeded 788 (Table 4). Estimates of *N*_*e*_ from MLNe ranged from 1686 to 3818 but confidence intervals were only finite for the estimate based on genetic changes from 1993–2001. LDNe provided point estimates for the samples from 2001 to 2009 (2513 and 3365 respectively) but confidence intervals in all years included infinity. ONeSAMP estimated *N*_*e*_ based on data only from 1993 (*N*_*e*_ = 1256 CI: 788–2644). Across all methods, all *N*_*e*_ estimates exceeded 1000, and 5 of the 9 non‐infinite estimates were between 1256 and 2006. *N*_*e*_/*N*_*c*_ ratios obtained from ONeSAMP, LDNe, MLNe and *F*_*TEMP*_ varied widely from 0.09 to 1.35 across the time periods considered (Table 4). Several of the *N*_*e*_ estimates resulted in an *N*_*e*_/*N*_*c*_ ratios that exceeded 1.0. Although theoretically possible if variance in reproductive success is non‐existent or less than random expectation (Charlesworth 2009), this is almost certainly implausible, especially given the fact that we know that family correlated marine survival in this populations may exceed Poisson variance in reproductive success (Geiger et al. 1997, 2007). Therefore, these values are almost certainly due to the large uncertainty surrounding these fairly large *N*_*e*_ estimates.

**Table 4:** Estimates for the genetic effective population size *N*_*e*_ and the *N*_*e*_/*N*_*c*_ ratio. Values in parentheses are the lower and upper 95% confidence/credible intervals. For the temporal methods (*F*_*TEMP*_ and MLNe), the 1993 value refers to the time period 1993–2001, the 2001 value refers to 2001–2009 and 2009 refers to 1993–2009

		*N*_*E*_	
Method	1993	2001	2009
ONeSAMP	1256 (788, 2644)	∞	∞
LDNe	∞	2513 (1182, ∞)	3365 (1148, ∞)
*F*_*TEMP*_	1598 (844, 6005)	1473 (836, 3938)	4962 (2128, ∞)
MLNe	2006 (1041, ∞)	1686 (963, 5039)	3818 (2113, ∞)

		*N*_*E*_/*N*_*C*_	
Method	1993	2001	2009
ONeSAMP	0.215	NA	NA
LDNe	NA	0.09	1.14
*F*_*TEMP*_	0.57	0.21	1.35
MLNe	0.71	0.235	1.04

### Inter‐annual changes in genetic divergence and N_e_

Generally, there did not appear to be strong evidence for any inter‐annual changes in genetic divergence across the migration timing distribution from 1993 to 2009 (Fig. 2). There were, however, five pairs of estimates for the autocorrelation coefficient that did not have overlapping 95% confidence intervals in different years (i.e. the strength of genetic correlation between individuals migrating the same number of days apart from one another varied in different years). The estimate of the correlation for fish migrating within 4 days of each other in 1993 was smaller (*r* *=* 0.0054 CI: 0.0016–0.0091) than that for 2001 (*r* = 0.0125 CI: 0.0095–0.0154), and the 95% confidence intervals for 2009 barely overlapped (*r =* 0.0125 CI: 0.0091–0.0160) with the estimate from 1993, suggesting that positive autocorrelation for fish migrating within 4 days of one another was weaker in 1993. Additionally, three of the non‐overlapping estimates were higher than expected positive values of the autocorrelation coefficient (relative to the associated negative value in a different year) for which we have no biological explanation. The weaker patterns of divergence in 1993 were also evident from the *G*‐tests for differences in allele frequencies between groups of individuals sampled in different quartiles of the migration timing distribution (Table 3).

The 95% confidence intervals for *G”*_*ST*_ in each year overlapped with the 95% confidence intervals for *G”*_*ST*_ for every other year; therefore, we were unable to detect differences in the strength of genetic divergence by migration timing in different years from this summary statistic. This does not support the prediction that there has been a decrease in genetic structure by migration timing due to significant changes in the variance and central tendency of adult migration timing into freshwater. Similarly, the 95% confidence/credible intervals for the point estimates of *N*_*e*_ from ONeSAMP, LDNe, MLNe and *F*_*TEMP*_ overlapped across all time periods (where estimates were available).

## Discussion

In this study we used temporal genetic data for a pink salmon population to test for genetic differences between early‐ and late‐migrating fish, to determine whether circadian rhythm genes appear to be related to migration timing, and to estimate the genetic effective population size and the ratio between the genetic effective population size and abundance. We used our temporal data to test our hypothesis that rapid changes in migration timing in this population have altered patterns of genetic diversity. Similar to what has been found in other salmonid populations, we observed that Auke Creek pink salmon demonstrate genetic differences between fish that migrate into freshwater at different times. However, the magnitude of divergence was small and did not result in any distinct population grouping based on allele frequencies. Circadian rhythm genes were the only loci that showed any evidence of divergent selection between early‐ and late‐migrating fish, but patterns of selection were inconsistent across years. Across all years, our estimates of *N*_*e*_ and the *N*_*e*_/*N*_*c*_ were quite large and generally greater than values observed in other populations, but were hampered with imprecision. Despite rapid changes toward earlier migration timing and loss of phenotypic variation, patterns of within‐population genetic divergence based on allozyme (1979, 1981, 1983) and microsatellite data (1993, 2001, 2009) have remained relatively stable. Similarly, the genetic effective population size appears stable from 1993 to 2009. Below, we provide potential explanations for these observations and discuss their implication for the management and conservation of genetic diversity.

### Intra‐population genetic differentiation

Kovach et al. (2012a) noted that there was a selection event against very late‐migrating fish from 1989 to 1993, which caused a loss of genetic structure at an experimental genetic marker for late migration timing. At the microsatellite loci examined in this paper, we observed little evidence of genetic divergence due to migration timing in 1993 (one to two generations after this event), and reduced positive genetic correlation for individuals migrating within 4 days of one another. But, the data from 2001 to 2009 confirmed the existence of genetic divergence between early‐ and late‐migrating fish that was also observed with allozyme data in the late 1970s and 1980s (McGregor 1983; McGregor et al. 1998; Gharrett et al. 2001). Whether the lack of divergence in 1993 was due to the selective event against very late‐migrating fish that occurred from 1989 to 1993 is unclear. If so, this suggests that climate‐induced selective events may lead to short‐term changes in neutral genetic structure, but general patterns, at least in this instance, re‐emerged.

Alternatively, it may be more difficult to detect subtle divergence in some years than in others. For example, intra‐annual environmental variation (e.g. stream flow) may cause individuals from different portions of the migration timing distribution to migrate earlier or later, resulting in overlaps in migration timing and a mixture of early‐ and late‐spawning fish in the genetic samples collected. This possibility is supported by the fact the number of days over which fish migrated into Auke Creek in 1993 was the lowest on record for this population. Alternatively, biological phenomena such as strong assortative mating, and/or reduced fitness of progeny from mating events between individuals with different migration timing are acting within this population. Therefore, sampling multiple generations may be required to detect genetic divergence based on variation in migration/reproductive timing. Another explanation is that migration from outside populations has helped re‐establish the genetic differentiation between early‐ and late‐migrating fish in this population (i.e. late‐migrating fish are from different populations). This possibility seems unlikely because gene flow and migration between Auke Creek and other nearby populations is relatively low (Gharrett et al. 2001; Gilk et al. 2004), and nearby populations do not appear to migrate as late in the season (mid‐ to late‐September) as the latest migrating fish in Auke Creek (AJ Gharrett *personal observation*).

Interestingly, no temporal changes were detected in estimates of *G”*_*ST*_ between early‐ and late‐migrating fish from 1979 to 2009, suggesting genetic stability has been present over 16 generations. Compared to the other methods used for the microsatellite data in this study (homogeneity and autocorrelation) and the allozyme data in previous studies (likelihood ratio tests; McGregor et al. 1998), *G”*_*ST*_ was less sensitive (i.e. unable to detect differentiation). Therefore, it is possible that we failed to detect very subtle temporal changes in population structure because we failed to detect any genetic divergence with *G”*_*ST*_. Unfortunately, this was the best method available to compare divergence between the allozyme allele frequency data from 1979, 1981, and 1983, and the microsatellite data we collected from samples in 1993, 2001, and 2009.

### Circadian rhythm genes and migration timing

Genetic differentiation at a *Cryptochrome* gene (*Cry2b*) exceeded neutral expectation in two of the 3 years for which we had data, indicating that this gene may be associated with or linked to genes that influence migration timing. Importantly, genetic variation was extremely low in the polyQ repeat region of the *Clock* gene (*OtsClock1b*) and *Cry2b* (Fig. 3). The polyQ repeat region of *Clock* is related to migration timing in various taxa from fish (O'Malley and Banks 2008; O'Malley et al. 2010a) to birds (e.g. Liedvogel et al. 2009; Caprioli et al. 2012). Other studies on birds have noted extremely low levels of genetic variation at this particular locus, and researchers have argued that this may result from selection favoring one particular allele (Liedvogel and Sheldon 2010; Dor et al. 2011). Similarly, the lack of variation at this locus within pink salmon (also see O'Malley et al. 2010a,b) may be due historic selection for the most abundant allele. Also, the genetic variation at *Cry2b* was low and,its outlier status could result from the random occurrence of a few individuals with alternate alleles. Interestingly though, *Cry2b* also demonstrated the strongest genetic changes over time (across generations) of any of these loci (Kovach et al. 2012a), but those changes did not exceed neutrality. Altogether, these are intriguing results, but research focused on more generations or on different populations will be needed to resolve if *Cry2b* plays any role in pink salmon migration timing. On the other hand, the results from this study and the inter‐generational tests for selection in Kovach et al. (2012a) do not provide evidence that *OtsClock1b* and *Cry3* mediate migration timing in this population.

### Genetic effective population size

Our point estimates of *N*_*e*_ from *F*_*TEMP*_, MLNe, LDNe, and ONeSAMP were all in excess of 1200 and the lowest confidence/credible interval was 788. These estimates are considerably larger than the median *N*_*e*_ of 267 reported in a recent meta‐analysis examining *N*_*e*_ across all species and taxa (Palstra and Ruzzante 2008). Similarly, all but one *N*_*e*_/*N*_*c*_ estimate exceeded the median estimate of 0.14 reported in the same study, and the mean value 0.11 from Frankham (1995b). Several of our estimates were, however, quite close to the recently updated and corrected median *N*_*e*_/*N*_*c*_ value of 0.23 in Palstra and Fraser (2012). For pink salmon in this population, family‐correlated marine survival reduces *N*_*e*_/*N*_*c*_ to approximately 0.5 (Geiger et al. 1997, 2007). Fluctuating abundance and larger than Poisson variance in reproductive success occurring during reproduction and early freshwater development appear to further reduce *N*_*e*_/*N*_*c*_ by nearly 0.25. Importantly, five of the nine *N*_*e*_/*N*_*c*_ estimates based on finite estimates exceeded the empirical baseline ceiling of 0.5 based on family correlated marine survival obtained in previous studies of this population. Although migration/gene flow between Auke Creek and other nearby locations is relatively limited, it certainly occurs, and these larger than expected *N*_*e*_ values might be due to gene flow from outside populations. Several recent papers on salmon populations have demonstrated that ignoring gene flow can induce an upward bias in *N*_*e*_ estimates (Palstra et al. 2009; Palstra and Ruzzante 2011). Regardless, it is clear that *N*_*e*_ is quite large in this population. Since *N*_*e*_ depends largely on habitat availability in salmonid fish (Shrimpton and Heath 2003; Palstra et al. 2009; Ozerov et al. 2012), our data indicate that pink salmon in relatively small coastal stream systems such as Auke Creek can have substantial effective population sizes. Generally, our results are more congruent with *N*_*e*_ estimates for salmonid species with large abundances and increased gene flow (Gomez‐Uchida et al. 2013).

Contrary to our prediction that *N*_*e*_ may have decreased due to selection against late migrating fish and/or as a result of increased competition during spawning, we did not detect a trend toward decreasing values of *N*_*e*_ from 1993 to 2009 (eight complete generations). While this strongly suggests that *N*_*e*_ has not rapidly decreased in this population (Antao et al. 2010), the ability to detect small changes in *N*_*e*_ for this population is limited by low statistical power at these effective sizes (i.e. >1000; Palstra et al. 2009; Waples and Do 2010). From a biological standpoint, there is strong competition (density dependence) for adult spawning sites in Auke Creek pink salmon, and this competition leads to redd superimposition (the destruction of salmon eggs due to one fish spawning on anther fishes nest; Fukushima et al. 1998). Thus, the stability in *N*_*e*_ may be due to the decline in the late‐migrating phenotype and therefore a decrease in the number of redds of early‐spawning fish that are destroyed by late‐spawning fish. Alternatively, genetic compensation (e.g. Ardren and Kapuscinski 2003), may also explain the lack of evidence for a change in *N*_*e*_; though there are fewer late spawning fish, the variability in their reproductive variance may be diminished because of reduced competition during spawning. Indeed, genetic compensation is common in salmonid fish (Palstra and Ruzzante 2008).

## Conclusion

Understanding the factors that limit or decrease genetic diversity within populations will improve our understanding of adaptive potential and, therefore, persistence in the face of climate change (Frankham 2005; Kinnison and Hairston 2007). In salmonid fishes, the importance of phenotypic variation (presumably due in part to genetic variation) for population stability during environmental change is well documented (Hilborn et al. 2003; Greene et al. 2010; Schindler et al. 2010) highlighting the need to understand mechanisms influencing genetic diversity both within and between populations. Despite the proliferation of studies demonstrating climate‐induced phenological shifts, it is unclear how these shifts influence genetic diversity (Heard et al. 2012). We focused on a single population, but changes in phenology can also influence the distribution of genetic variation across populations if they affect interactions among populations and the probability of gene flow (Franks and Weiss 2009; Rossetto et al. 2011). Surprisingly, patterns of genetic diversity in Auke Creek pink salmon are stable and have been resilient to rapid phenological and environmental changes, including a 2‐week shift in migration timing. While future research on the impacts of phenological changes on genetic diversity is needed (Heard et al. 2012; Pauls et al. 2012), climate‐induced changes in spatial distribution have proven or are predicted to have substantial impacts on genetic diversity (e.g. Alsos et al. 2012; Rubidge et al. 2012). Given our current (limited) knowledge, conservation and management actions concerned with protecting genetic diversity during future climate warming may be most effective if focused on the potential consequences of distributional as opposed to phenological shifts.

## Data archiving statement

Data deposited in the Dryad repository: doi:10.5061/dryad.p2m22.
